# Intersection of Nativity and English Proficiency With Receipt of Person-Centered Contraceptive Counseling

**DOI:** 10.1097/og9.0000000000000013

**Published:** 2024-06-13

**Authors:** Emily R. Boniface, Katherine Courchaine, Katie Hansen, Blair G. Darney

**Affiliations:** Oregon Health & Science University, and the OHSU-Portland State University School of Public Health, Portland, Oregon; and Centro de Investigación en Salud Poblacional (CISP), Instituto Nacional de Salud Pública (INSP), Cuernavaca, Mexico.

## Abstract

**OBJECTIVE:**

To assess disparities in receipt of person-centered contraceptive counseling among a nationally representative sample of U.S.- and foreign-born individuals with differing English proficiency.

**METHODS:**

We conducted a secondary analysis of the 2017–2019 wave of the National Survey of Family Growth and included female respondents aged 15–49 years who received contraception services in the previous 12 months and provided responses to all four items of the Person-Centered Contraceptive Counseling measure. We categorized respondents into four groups: 1) U.S.-born individuals who speak English very well, 2) foreign-born individuals who speak English very well, 3) U.S.-born individuals who speak English less well, and 4) foreign-born individuals who speak English less well. We created an adjusted logistic regression model, with receipt of person-centered contraceptive counseling as the outcome and the four nativity–English proficiency groups as the primary independent variable, then calculated the predicted probability of receiving person-centered contraceptive counseling for each group, adjusted for age, income, urban or rural status, and parity as covariates. We also assessed the four Person-Centered Contraceptive Counseling items individually.

**RESULTS:**

Our final study sample included 2,221 respondents (weighted N=26,531,058). Respondents who spoke English very well had comparable adjusted predicted probabilities of reporting person-centered counseling regardless of nativity (52.2% [95% CI, 48.5–55.9%] for U.S.-born individuals and 50.6% [95% CI, 38.2–62.9%] for foreign-born individuals). Among respondents who spoke English less well, U.S.-born individuals had a lower adjusted probability of reporting person-centered counseling than foreign-born individuals (31.0% [95% CI, 13.5–48.5%] vs 40.6% [95% CI, 29.2–52.0%], respectively). The largest disparities were for the items related to letting the respondent say what mattered most to them about their birth control and taking their preferences about their birth control seriously.

**CONCLUSION:**

Person-centered contraceptive counseling was lower for respondents with limited English proficiency, particularly among U.S.-born individuals; English proficiency mitigated disparities by nativity. Clinicians should prioritize person-centered contraceptive care, especially for patients with limited English proficiency.

## Abstract 2

Receipt of person-centered contraceptive counseling is lower for respondents with limited English proficiency; English proficiency mitigated disparities by nativity.

Patient-centeredness is a key domain of health care quality,^1^ and merits special focus in reproductive health care in light of historical and current policies and practices that violate patient autonomy, including forced sterilization and contraceptive coercion.^2–5^ The Person-Centered Contraceptive Counseling measure was developed to assess patient experience of care, specifically patient-centeredness, when discussing contraception with their clinician.^6,7^ The Person-Centered Contraceptive Counseling measure is a complement to claims based measures of contraception access^8^ and was endorsed by the National Quality Forum in 2020.^9^

Disparities in contraceptive care quality by factors such as race, ethnicity, and income have been well documented,^10–12^ and recent work using the Person-Centered Contraceptive Counseling measure has specifically examined differences in contraceptive counseling quality associated with patient characteristics, including both nativity (ie, whether or not a person was born in the United States) and English proficiency.^13,14^ Both nativity and English proficiency can play a crucial role in how individuals are able to access and navigate the U.S. health care system and the quality of the care they receive.^15–19^ However, these factors are interrelated, and analyses that examine them separately may obscure important differences based on patients' lived experiences.

The purpose of this study was to assess disparities in receipt of person-centered contraceptive counseling among a nationally representative sample of U.S.- and foreign-born individuals with differing English proficiency. We hypothesized that U.S.-born individuals with high English proficiency would report the highest levels of person-centered care, and foreign-born individuals with limited English proficiency would report the lowest levels.

## METHODS

We conducted a secondary analysis of public use data from the 2017–2019 wave of the National Survey of Family Growth, the first wave in which the Person-Centered Contraceptive Counseling measure was included. The National Survey of Family Growth is a nationally representative survey conducted by the National Center for Health Statistics to assess reproductive health outcomes such as birth, pregnancy, fertility, and contraception use.^20^ The survey samples noninstitutionalized men and women of reproductive age using a stratified, multistage probability sample,^21^ and has a response rate of approximately 69%. Data are collected through in-person interviews in English or Spanish, with private self-administration for questions about sensitive topics. Since 2011, interviews have been conducted continuously, with data released every 2 years.

Our study sample included respondents aged 15–49 years who were identified as female (by themselves or the household member who completed the household screener questionnaire) and provided responses to the Person-Centered Contraceptive Counseling measure. The National Survey of Family Growth uses a binary definition of gender; as such, we use the term female throughout while acknowledging that this may not reflect all respondents' gender identities. To be asked about the Person-Centered Contraceptive Counseling measure, respondents must have received at least one of seven contraception-related services in the previous 12 months (all wording is from the National Survey of Family Growth): a method of birth control or a prescription for a method, counseling or information about birth control, a check-up or medical test related to using a birth control method, emergency contraception or a prescription for it, counseling or information about emergency contraception, a sterilizing operation, or counseling or information about getting sterilized.

**Fig. 1.**
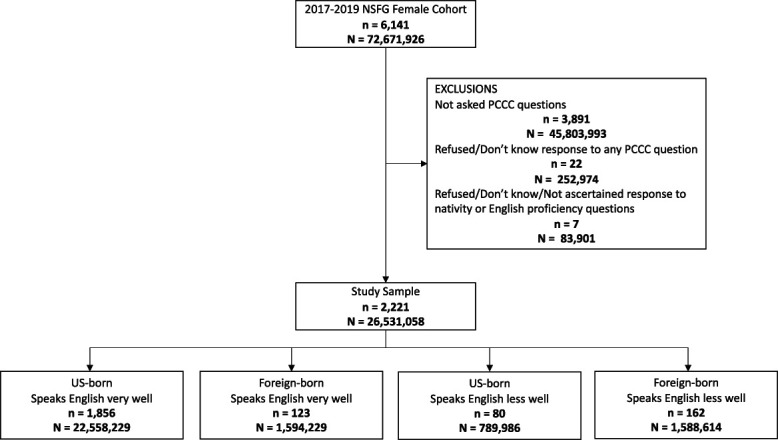
Study flow diagram, with unweighted (n) and survey-weighted (N) respondent counts. NSFG, National Survey of Family Growth; PCCC, person-centered contraceptive counseling.

Our primary outcome was receipt of *person-centered contraceptive counseling*, defined as a rating of “excellent” on all four of the Person-Centered Contraceptive Counseling measure questions.^22^ Respondents were asked to rate their contraceptive services clinician on four separate items: 1) “respecting me as a person,” 2) “letting me say what mattered to me about my birth control,” 3) “taking my preferences about my birth control seriously,” and 4) “giving me enough information to make the best decision about my birth control.” Responses ranged from 1 (“poor”) to 5 (“excellent”). We followed guidance from the developers of the Person-Centered Contraceptive Counseling measure, which recommends converting the responses into a binary measure: whether respondents rated their clinician as excellent on all four questions (yes or no).^22^ We excluded respondents with “refused” or “don't know” responses to any of the four Person-Centered Contraceptive Counseling questions (unweighted n=22; Fig. 1).

*Boniface. Nativity, Language, and Person-Centered Care. O&G Open 2024*.

Our primary independent variable was an interaction between respondent nativity and English proficiency, although we also considered both variables separately. Nativity is classified in the National Survey of Family Growth as being born within or outside the United States, which we identified as U.S.-born or foreign-born. Respondents reported their ability to speak English as “very well,” “well,” “not well,” or “not at all,” which we collapsed into a binary variable (speaks English very well vs less well) due to the small number of respondents with lower levels of proficiency. With these two binary measures, we created four groups: 1) U.S.-born individuals who speak English very well, 2) foreign-born individuals who speak English very well, 3) U.S.-born individuals who speak English less well, and 4) foreign-born individuals who speak English less well. Our study sample excluded respondents without known nativity or English proficiency (unweighted n=7).

We also included several sociodemographic variables in our analysis. For descriptive analyses, we categorized respondent age at the time of survey into 5-year bands: 15–19, 20–24, 25–29, 30–34, 35–39, 40–44, and 45–49 years; we included age as a continuous variable in our regression model. We classified race and ethnicity as Latina (regardless of reported race or races), non-Latina Black (Black), non-Latina White (White), and non-Latina some additional race. We created our categories using the recoded race variable in which multiracial respondents identify the race they said best described them. The “some additional race” category included American Indian or Alaska Native, Asian, and Native Hawaiian or Pacific Islander respondents; disaggregated categories are not available in the National Survey of Family Growth public-use data. We included race and ethnicity in our study to capture exposure to structural racism and race-based clinician bias. We identified respondents' country of origin as the United States, Mexico, other Latin American country, or some other country; disaggregated information on respondents’ countries of origin is not available in the public-use data. We categorized education level as less than high school or equivalency certificate, high school or equivalency certificate, some college, or 4 years of college or more. We created four income categories based on the federal poverty level: less than 100%, 100–199%, 200–299%, or 300% of the federal poverty level or more. We classified health insurance status as private insurance, public insurance, or uninsured. We created binary variables for relationship status (married or cohabitating: yes or no), urban rural status (urban or suburban vs rural), and parity (nulliparous vs parous).

All analyses incorporated the poststratified complex survey weights released by the National Survey of Family Growth, which account for the multilevel sampling design as well as nonresponse.^23,24^ We tabulated all sociodemographic variables, overall and by our four nativity–English proficiency groups. We calculated the percent of respondents who reported the receipt of person-centered contraceptive counseling and created bar graphs to visualize these percentages, by nativity alone, by English proficiency alone, and by our four nativity and English proficiency groups. We then created both unadjusted and adjusted logistic regression models and corresponding plots of the odds ratios, with receipt of person-centered contraceptive counseling as the outcome and the four nativity–English proficiency groups as the primary independent variable. The adjusted model also included age, income, urban or rural status, and parity as covariates; due to the relatively small sample size of some of our nativity–English proficiency groups we were limited in our ability to adjust for all potential confounders. To improve the interpretability or our modeling results, we calculated the predicted probability of receiving person-centered contraceptive counseling for each nativity–English proficiency group by using average marginal effects from the adjusted model. We also created separate bar graphs for each the four Person-Centered Contraceptive Counseling questions depicting the percentage of each person in the nativity–English proficiency group who rated their clinician as excellent. We performed a sensitivity analysis that incorporated inverse propensity score weighting to address potential confounding by differences between our nativity–English proficiency groups^25^; the propensity score model included all covariates in our adjusted model. All analyses were conducted in Stata 17.0. The study was approved as nonhuman subjects research by the Oregon Health & Science University IRB (STUDY00025628).

## RESULTS

The 2017–2019 wave of the National Survey of Family Growth included 2,250 respondents who received contraceptive services in the previous year and thus were asked the Person-Centered Contraceptive Counseling questions (Fig. 1). After excluding 3,891 respondents who did not receive contraceptive services in the previous year and 29 respondents due to missing responses for the Person-Centered Contraceptive Counseling items (n=22) or nativity and English proficiency questions (n=7), our final study sample included 2,221 respondents (weighted N=26,531,058). The majority of respondents (85.0% weighted) were U.S.-born individuals who spoke English very well; the two foreign-born groups each made up 6.0% of the sample, and the U.S.-born individuals who spoke English less well comprised 3.0% of the sample.

U.S.-born respondents skewed younger than those born outside of the United States (Table 1). Among respondents who spoke English very well, the majority (67.8%) of the U.S.-born individuals were White, whereas the foreign-born individuals were fairly evenly distributed across the four race and ethnicity groups. Among respondents who spoke English less well, the U.S.-born individuals were largely Latina and Black (63.5%), whereas the majority of the foreign-born individuals were Latina (72.8%). Foreign-born respondents who spoke English less well were primarily from Mexico or other Latin American countries (72.7%), and those who spoke English very well were largely from other parts of the world (70.9%). Respondents who spoke English very well were more likely to have a college degree, higher income, and private health insurance than those who spoke English less well, regardless of nativity. Among respondents who spoke English less well, U.S.-born individuals were more likely to have lower educational attainment (41.9% had less than a high school education vs 36.6% of foreign-born individuals), and foreign-born individuals were more likely to be uninsured (30.6% vs 6.9% of U.S.-born individuals).

**Table 1.** Demographic Characteristics of Female Survey Respondents Asked About Receipt of Person-Centered Contraceptive Counseling, by Nativity and English Proficiency, 2017–2019 National Survey of Family Growth*

Characteristic	Speaks English Very Well		Speaks English Less Well		Overall	*P*
	U.S.-Born	Foreign-Born	U.S.-Born	Foreign-Born		
Unweighted n	1,856	123	80	162	2,221	
Weighted N	22,558,229	1,594,229	789,986	1,588,614	26,531,058	
Age group (y)						.021
15–19	15.4	6.5	22.5	7.2	14.6	
20–24	21.9	22.3	17.4	8.6	21.0	
25–29	20.9	18.3	25.2	17.3	20.7	
30–34	17.3	25.0	25.3	20.5	18.2	
35–39	10.3	12.1	1.9	22.2	10.8	
40–44	7.7	6.4	7.1	17.7	8.2	
45–49	6.5	9.4	0.6	6.6	6.5	
Race and ethnicity						—†
Latina	13.1	29.1	39.5	72.8	18.4	
Non-Latina Black	15.6	21.2	24.0	2.7	15.4	
Non-Latina some additional race‡	3.6	25.1	16.9	20.8	6.3	
Non-Latina White	67.8	24.6	19.5	3.8	59.9	
Country of origin						—†
United States	100.0	0.0	100.0	0.0	88.0	
Mexico	0.0	14.4	0.0	49.6	3.8	
Other Latin American	0.0	14.7	0.0	23.1	2.3	
Other	0.0	70.9	0.0	27.3	5.9	
Education						<.001
Less than high school or equivalency certificate	12.5	5.8	41.9	36.6	14.5	
High school or equivalency certificate	18.3	8.9	27.2	23.8	18.3	
Some college	32.3	34.3	20.5	15.8	31.0	
4-y college or more	36.9	51.0	10.4	23.8	36.2	
Federal poverty level (%)						<.001
Less than 100	18.6	16.5	44.3	43.3	20.7	
100–199	21.1	17.3	13.2	27.3	21.0	
200–299	18.0	14.9	30.1	11.0	17.8	
300 or more	42.2	51.3	12.4	18.5	40.5	
Insurance status						<.001
Private	71.5	67.3	38.4	33.7	68.0	
Public	22.1	22.7	54.7	35.7	23.9	
Uninsured	6.4	10.0	6.9	30.6	8.1	
Married or cohabitating	47.5	51.6	49.0	68.9	49.1	.055
Rural residence	16.2	8.2	18.2	14.4	15.7	.520
Parous	42.1	50.9	59.8	77.0	45.2	<.001

Data are % unless otherwise specified.

* Excludes respondents without known responses to the Person-Centered Contraceptive Counseling, nativity, or English proficiency survey questions. Column percentages may not sum to 100% due to rounding.

† Hypothesis test not performed.

‡ Respondents identified as American Indian or Alaska Native, Asian, or Native Hawaiian or Pacific Islander.

**Fig. 2.**
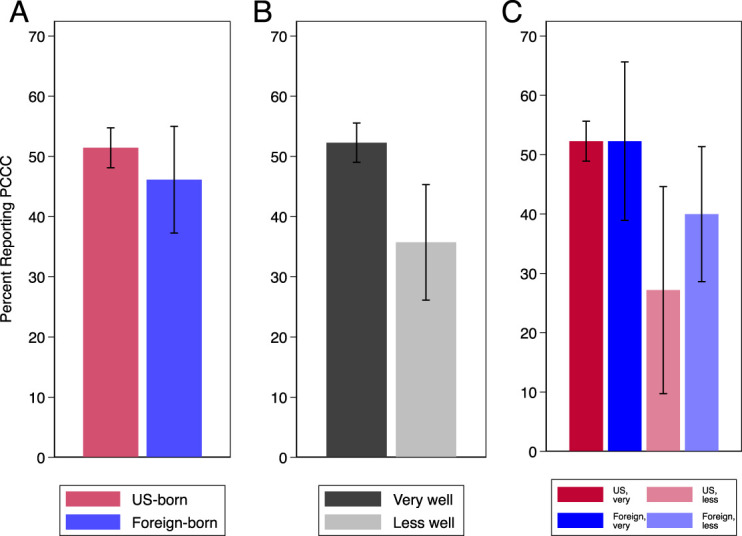
Weighted percentage of 2017–2019 female National Survey of Family Growth respondents reporting receipt of person-centered contraceptive counseling, by nativity (**A**), English proficiency (speaks English very well vs less well) (**B**), and nativity and English proficiency (**C**) (n=2,221). *Error bars* represent 95% CIs. Excludes respondents without known responses to the Person-Centered Contraceptive Counseling (PCCC), nativity, or English proficiency survey questions.

We first examined the unadjusted associations between receipt of person-centered contraceptive counseling and nativity and English proficiency separately. Respondents who were born in the United States were more likely than the foreign-born respondents to report receiving person-centered contraceptive counseling (51.4% [95% CI, 47.6–55.2%] vs 46.1% [95% CI, 37.0–55.6%], respectively; Fig. 2A), as were those who spoke English very well compared with those who spoke English less well (52.3% [95% CI, 48.3–56.2%] vs 35.7% [95% CI, 27.7–44.7%], respectively; Fig. 2B).

*Boniface. Nativity, Language, and Person-Centered Care. O&G Open 2024*.

We then assessed the unadjusted association between receipt of person-centered contraceptive counseling and the interaction between nativity and English proficiency (Fig. 2C). Among respondents who spoke English very well, the unadjusted prevalence of reporting person-centered care was identical for both nativity groups (52.3%; U.S.-born individuals: 95% CI, 48.4–56.1%; foreign-born individuals: 95% CI, 37.6–66.6%). Among respondents who spoke English less well, the proportion reporting person-centered care was smaller than for those with higher English proficiency, and U.S.-born respondents were less likely to report receiving person-centered contraceptive counseling than those born outside the United States (27.2% [95% CI, 14.1–46.0%] vs 40.0% [95% CI, 29.2–51.9%], respectively).

**Fig. 3.**
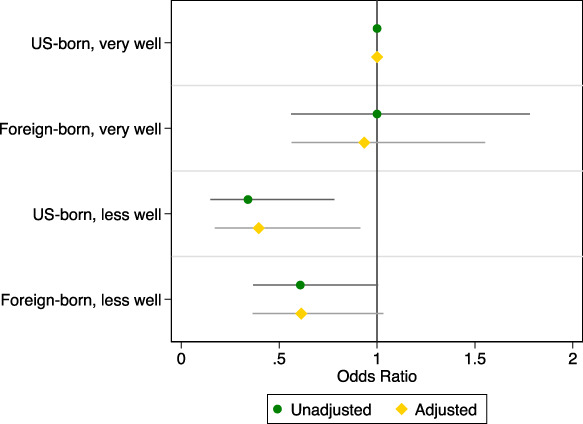
Unadjusted and adjusted odds ratios of receiving person-centered contraceptive counseling among 2017–2019 female National Survey of Family Growth respondents (n=2,221), by nativity and English proficiency. English proficiency was categorized as speaks English very well vs less well; the referent group is U.S.-born respondents who speak English very well. Adjusted estimates include respondent age, federal poverty level, urban or rural status, and parity. Excludes respondents without known responses to the Person-Centered Contraceptive Counseling, nativity, or English proficiency survey questions.

After adjusting for respondent sociodemographics, the pattern across our nativity and English proficiency groups remained similar (Fig. 3). Respondents who spoke English very well had comparable predicted probabilities of reporting person-centered counseling, regardless of nativity (52.2% [95% CI, 48.5–55.9%] for U.S.-born individuals and 50.6% [95% CI, 38.2–62.9%] for foreign-born individuals. Among respondents who spoke English less well, the adjusted predicted probabilities were slightly higher than the unadjusted percentages, but U.S.-born individuals still had a lower probability of reporting person-centered counseling than foreign-born individuals (31.0% [95% CI, 13.5–48.5%] vs 40.6% [95% CI, 29.2–52.0%], respectively). Higher income, rural residence, and parity were all associated with higher odds of reporting receipt of person-centered counseling; full adjusted model results can be found in Appendix 1, available online at http://links.lww.com/AOG/D719.

*Boniface. Nativity, Language, and Person-Centered Care. O&G Open 2024*.

**Fig. 4.**
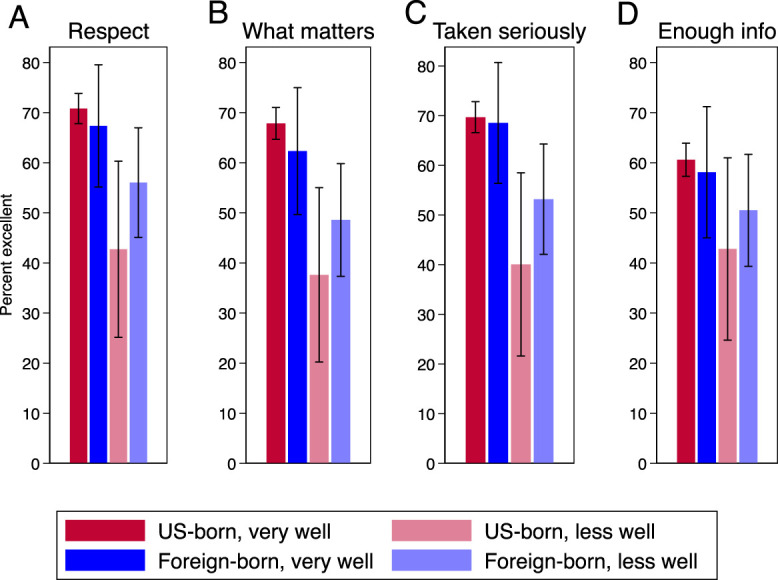
Percentage of 2017–2019 female National Survey of Family Growth respondents reporting receipt of excellent care for each of the Person-Centered Contraceptive Counseling questions: how the clinician rated on respecting me as a person (labeled “Respect”) (**A**), letting me say what mattered to me about my birth control (“What matters”) (**B**), taking my preferences about my birth control seriously (“Taken seriously”) (**C**), and giving me enough information to make the best decision about my birth control (“Enough info”) (**D**). Weighted estimates are presented by nativity and English proficiency (n=2,221). *Error bars* represent 95% CIs. English proficiency was categorized as speaks English very well vs less well. Excludes respondents without known responses to the person-centered contraceptive counseling, nativity, or English proficiency survey questions.

The overall patterns across our nativity and English proficiency groups for the individual Person-Centered Contraceptive Counseling items remained consistent (Fig. 4A–D). The two groups of people who spoke English very well were similar and consistently scored higher than those who spoke English less well; among those who spoke English less well, U.S.-born individuals always reported the lowest percentage of excellent scores. The largest disparities were for the items related to letting the respondent say what mattered most to them about their birth control (Fig. 4B) and taking their preferences about their birth control seriously (Fig. 4C). Our sensitivity analysis incorporating inverse propensity score weighting did not alter our findings in a meaningful way.

*Boniface. Nativity, Language, and Person-Centered Care. O&G Open 2024*.

## DISCUSSION

Using a nationally representative sample of reproductive-aged females, we found that receipt of person-centered contraceptive counseling did not differ by nativity among respondents with high English proficiency. However, among respondents with more limited English proficiency, reports of receiving person-centered contraceptive counseling were lower than those with higher proficiency and were lowest for those born in the United States. After adjusting for respondent sociodemographics, differences driven by English proficiency were smaller, but overall trends remained the same. Among the individual Person-Centered Contraceptive Counseling items, disparities were largest for letting me say what mattered to me about my birth control and taking my preferences about my birth control seriously.

We found no difference in reported person-centeredness of care between nativity groups with high English proficiency. Language barriers can adversely affect an individual's ability to navigate the health care system as well as patient comprehension once care is accessed.^17,26–28^ Our results suggest that the ability to communicate effectively with a medical clinician can mitigate the negative experience of care quality among foreign-born respondents, and underscore the importance of examining the complexity of the immigrant experience, beyond simply nativity. Foreign-born respondents in our study sample were diverse with respect to their race and ethnicity, educational attainment level, and insurance status, which all differed substantially by English proficiency. Specifically, foreign-born individuals with high English proficiency were more likely to have a college education and private health insurance, which likely contributed to their reports of receiving higher quality contraceptive care than their counterparts with more limited English proficiency.

We hypothesized that foreign-born respondents with limited English proficiency would report the lowest rates of receiving person-centered contraceptive counseling. Instead, we found that U.S.-born respondents with limited English proficiency reported worse quality of care than their foreign-born counterparts. U.S.-born people with limited English proficiency are likely an especially marginalized group. They may have limited connections to dominant (English-speaking) society and face unique barriers navigating the health care system.^29,30^ Such marginalization may also increase their likelihood of living below the poverty limit, which is associated with lower probability of reporting high-quality care.^31^ The discrepancy may also be driven in part by racial discrimination or by clinician bias. The U.S.-born limited English proficiency group included the largest proportion of non-Latina Black respondents, who are the most likely to report negative health care experiences driven by racism and discrimination.^32^ There is also evidence for clinician bias based on many other forms of structural inequity, including socioeconomic status, age, gender, and marital status,^33–35^ all of which may contribute to reports of lower quality care in marginalized groups. Finally, it is possible that the difference between U.S.- and foreign-born respondents with limited English proficiency is driven by lower expectations of receiving person-centered care among the foreign-born individuals. Without measures of expectations of care quality, we are not able to evaluate how patient expectations may influence their reported Person-Centered Contraceptive Counseling outcomes, although previous literature suggests that expectations are directly tied to patient experience of care.^36,37^

In our nationally representative sample, even among the groups with the highest reported prevalence of person-centered contraceptive counseling, the overall percentages were just slightly higher than 50%. These findings are in keeping with previous work that used Person-Centered Contraceptive Counseling data from the National Survey of Family Growth,^13,14,31^ but the findings are slightly lower than data used to validate the Person-Centered Contraceptive Counseling measure.^7^ This may be due to differences in the study samples or due to the timing of data collection. In either case, it highlights the need to improve the person-centeredness of contraceptive counseling for a large proportion of contraceptive patients.

Our study has several strengths, including the use of nationally representative data, which allows population-based interpretation of our results. Previous studies that used the same data source have assessed language-related disparities in receipt of person-centered contraceptive counseling, but only among Hispanic respondents.^13,14^ By examining associations for all respondents with limited English proficiency, we are able to capture the experiences of all individuals who may experience lower quality care due to language barriers. In addition, our intersectional approach acknowledges the connection between nativity and English proficiency rather than treating them as separate phenomena.

Despite these strengths, our study has some limitations. First, the relatively small number of National Survey of Family Growth respondents who were asked the Person-Centered Contraceptive Counseling questions and the lack of diversity within that subsample limited our ability to draw statistically significant conclusions between our nativity and English proficiency groups. Larger data sets are needed to reliably identify disparities in person-centered care provision. Second, the lack of detailed data on foreign-born respondents' race and country of origin could obscure important differences between groups whose lived experiences are not shared. We are not able to address any differences in the quality of care received by respondents who do not identify as White, Black, or Latina, or who were born outside of the United States or a Latin American country. Third, although our classification of limited English proficiency (ie, whether or not a respondent speaks English very well) was consistent with standard definitions,^15^ we were not able to address differences among respondents with lower levels of English proficiency. Fourth, the National Survey of Family Growth does not include data on the use of interpreters during the contraceptive visit or the health care clinician delivering the counseling, so we were not able to account for the potential influence of interpreter services or clinician bias. Fifth, the public-use National Survey of Family Growth data do not include any information about the state or region of the respondent's residence, so we were unable to identify any geographic differences in receipt of person-centered care. Finally, although we used the most recent available data, they are still several years old. National Survey of Family Growth data collection was interrupted by the coronavirus disease 2019 (COVID-19) pandemic, and the next wave will not be released until after the 2022–2024 data-collection period.^38^ Although our results may not reflect current disparities in receipt of person-centered contraceptive counseling, they are based on the most recent nationally representative data available.

Receipt of person-centered contraceptive counseling differs by the patient's nativity and English proficiency. To address the disparities experienced by both U.S.-born and foreign-born individuals with limited English proficiency, provision of equitable and high-quality contraceptive care must be prioritized for all contraceptive patients.
